# Chromosome-Scale Genome Assembly and Transcriptome Assembly of Kawakawa *Euthynnus affinis*; A Tuna-Like Species

**DOI:** 10.3389/fgene.2021.739781

**Published:** 2021-09-20

**Authors:** Miloš Havelka, Eitaro Sawayama, Taiju Saito, Kazutoshi Yoshitake, Daiki Saka, Toshinao Ineno, Shuichi Asakawa, Motohiro Takagi, Rie Goto, Takahiro Matsubara

**Affiliations:** ^1^South Ehime Fisheries Research Center, Ehime University, Ainan, Japan; ^2^Department of Marine Science and Resources, College of Bioresource Sciences, Nihon University, Fujisawa, Japan; ^3^Laboratory of Aquatic Molecular Biology and Biotechnology, Graduate School of Agricultural and Life Sciences, The University of Tokyo, Tokyo, Japan; ^4^Aquaculture Research Institute, Kindai University, Shingu, Japan

**Keywords:** aquaculture, comparative genomics, eastern little tuna, genome annotation, linkage map, Thunnini

## Introduction

Kawakawa *Euthynnus affinis*, also known as eastern little tuna or mackerel tuna, is a species of tuna (Thunnini tribe, subgroup Pelagiaria clade) (Sanciangco et al., 2016). Tuna includes 15 species: eight of genus *Thunnus* (true tuna) and seven “tuna-like” of four genera: *Allothunnus, Auxis, Euthynnus*, and *Katsuwonus*.

*E. affinis* is widely distributed throughout the tropical and subtropical waters of the continental shelf areas of the Indo-Pacific region (Collette, 2001). The fish reaches a length of 45–60 cm and matures at approximately 3 years of age. It inhabits almost exclusively the upper layers of the ocean (Bernal et al., 2017) and feeds mainly on small pelagic fish (Griffiths et al., 2009). *E. affinis* makes up a substantial proportion of the commercial and artisanal fishery in many countries of the Indo-Pacific region. The meat of *E. affinis* is of high quality (Mukundan et al., 1979) with a comparatively high level of docosahexaenoic acid (Saito et al., 1999), but deteriorates rapidly if not properly handled (Mukundan et al., 1979). *E. affinis* exhibits the swimming mechanics of true tunas (Donley and Dickson, 2000) but has no swim bladder and differs from true tunas in red muscle distribution, allometry, and vascular anatomy (Bernal et al., 2017). The ability to maintain an elevated temperature in eye, brain, and red muscle has been suggested for the genus *Euthynnus* (Dickson et al., 2000), but reports specific to *E. affinis* are lacking.

Compared to true tunas, *E. affinis* has received scant attention from researchers, and little is known about its biology and physiology. This is likely to change, as *E. affinis* has recently become of interest in marine aquaculture. *E. affinis* is the second tuna species whose full-life cycle culture in captivity has been developed so far, including spawning, egg collection, incubation, larval rearing, and grow-out to marketable size (Yazawa et al., 2015, 2016).

Aquaculture in general is currently facing significant challenges to increasing production while maintaining sustainability (Bridson et al., 2020). Genetic improvement, *via* selective breeding and genetic engineering, is a major focus of research and can yield rapid benefits to efficient production in fish farming (Lu and Luo, 2020). To these ends, a high-quality species genome assembly is critical. Despite recent advances in sequencing technologies and genomics that, in addition to basic fish science (Lien et al., 2016; Hughes et al., 2018; Yuan et al., 2018; Du et al., 2020), have applications to aquaculture practices (Lu and Luo, 2020) and fisheries (Benestan, 2020), genomic information of tuna species is limited. To date, the genomes of only three tuna species are available in the public repositories, none of which are assembled to chromosome level. This situation exists within the entire Pelagiaria clade that, along with tuna, includes the economically important mackerel (Scombrini tribe) and bonito (Sardini tribe).

Here, we report the chromosome-level genome assembly of kawakawa *E. affinis* (NCBI:txid8227). To our knowledge, this is the first available chromosome-level assembly within the Pelagiaria clade. The reported genome assembly is accompanied by transcriptome assembly, genetic linkage map, annotation of transposons, repetitive elements, and 23,059 genes. The dataset provides a solid genome resource not only for further study of *E. affinis* basic biology and genome-scale selective breeding but also for enhancing both basic and applied research within the Pelagiaria clade.

## Materials and Methods

### Genome Sequencing and Assembly

The tissue sample was obtained from a single wild *E. affinis* female caught off the coast of Ainan, Ehime Prefecture, Japan. High-molecular-weight genomic DNA was isolated from fin using NucleoBond^®^ AXG columns with NucleoBond^®^ Buffer Set IV (Macherey-Nagel, Düren, Germany). The quantification of gDNA was performed by Quant-iT™ dsDNA Broad-Range Assay Kit (*Invitrogen*, Carlsbad, CA, USA), and molecular weight was estimated on 0.75% agarose gel by pulsed-field electrophoresis. The whole-genome sequencing library was prepared using Chromium Genome Library & Gel Bead Kit v. 2 (10x Genomics, Pleasanton, CA, USA) as described in the Chromium Genome Reagent Kit v. 2 User Guide. The library was sequenced on Illumina HiSeq X sequencing system using pair-end (2 × 150 bp) sequencing. The sequencing generated 920.3 million (M) reads of total 138.04 Gb with 93.8% and 87.7% of base having quality score Q > 30 in R1 and R2 reads, respectively.

Oxford Nanopore Technology (ONT) sequencing was used to obtain long reads for scaffolding. Libraries were generated using standard protocols from ONT with the SQK-LSK108 ligation sequencing kit (Oxford Nanopore Technologies, Oxford, United Kingdom). GridION X5 sequencing was performed according to the manufacturer's guidelines using three independent FLO-MIN107 (R9.5) flow cells. Base-calling was done by Albacore v. 1.2.4. (Oxford Nanopore Technologies, Oxford, United Kingdom). Raw reads were filtered by quality value QV9 (--minqual 9), and heads and tails were 50 bp trimmed each (--headtrim 50; --tailtrim50) in Yanagiba v. 1.0.0 (Taranto, 2017). The ONT sequencing generated 1.56 M reads with a total of 15.02 Gb, an average length of 9,650 bp, and N50 = 18,747 bp.

Prior to genome assembly, genome characteristics were estimated based on Illumina reads. Jellyfish v. 2.2.6 (Marçais and Kingsford, 2011) was applied to generate k-mer counting and frequency distributions of 19-, 21-, and 23-mers. Genome size, heterozygosity, and repeat content were estimated based on the generated k-mer count distributions using GenomeScope (Vurture et al., 2017) with high frequency k-mer cutoff = 10,000. The estimate in GenomeScope is based on an equation that models four evenly spaced negative binomial distributions of the k-mer profile to measure the relative abundances of heterozygous and homozygous, unique, and two-copy sequences (Vurture et al., 2017). The genome size estimate ranged from 745.82 Mb (*k* = 19) to 755.80 Mb (*k* = 23), heterozygosity rate was roughly estimated to be 0.67% (67 SNPs per 10 Kb), and repeat content estimate ranged from 137.00 Mb (*k* = 23) to 170.35 (*k* = 19; Supplementary Table 1).

Assembly of Illumina reads was performed in Supernova assembler v. 1.1.5 (10x Genomics, Pleasanton, CA, USA), with default parameters, except maximum reads (--maxreads), set at 386 M input reads to achieve 56 × raw coverage, as suggested in the Supernova protocol. Assembled scaffolds were loaded together with filtered ONT reads into PBJelly v. 15.8.24 (English et al., 2012) where identification of gaps >25 bp, gap filling, and scaffolding were performed using default parameters. In total, 11.31 Mb of gaps (34.8% of initial gap size) were successfully closed. The 787.60-Mb initial draft genome assembly was presented in pseudohaplotype format and consisted of 19,850 scaffold sequences (>1 Kb), of which 21.17 Mb (2.69%) represented unknown bases.

The redundancy of the genome assembly was reduced in three steps: First, 8,191 duplicated scaffolds (24.24 Mb) were removed using dedupe.sh from BBTools v. 38.87 (Bushnell, 2014). Furthermore, scaffolds < 2 Mb were clustered in CD-HIT v. 4.8.1 (Li and Godzik, 2006) with identity threshold ≥ 99% (-c 0.99) and word size -n 10. This step removed 1,044 scaffolds (5.1 Mb). Finally, retained scaffolds were self-aligned in LastZ v. 1.04 (Harris, 2007) with alignment identity threshold ≥ 99% (--identity = 99) and query coverage threshold ≥ 95% (--coverage = 95). If two different scaffolds were self-aligned, the longer one was retained and the shorter one was discarded. In total, 9,250 scaffolds (29.4 Mb; 3.7%) were removed from the initial assembly because of potential duplication or redundancy. The final scaffold-level assembly consisted of 10,600 scaf-folds of 758.20 Mb (19.89 Mb gaps), N50 = 24.78 Mb, and the longest scaffold = 35.13 Mb.

### Linkage Mapping and Chromosome-Level Scaffolding

We obtained two types of linkage evidence: diploid, based on full-sib family linkage analyses, and haploid, based on linkage analyses of interspecific hybrids (Yoshitake et al., 2018) of *E. affinis* female and *T. orientalis* male. Based on a genome coordinate of each marker in the linkage maps, we anchored and oriented scaffolds into pseudochromosomes.

To construct a diploid linkage map, DNA was extracted from fin clips of parents and 94 of their progeny using NucleoSpin^®^ Tissue (Macherey-Nagel, Düren, Germany). All specimens were assessed for body weight, standard length, head length, and body depth (Supplementary Table 2). Genotyping by random amplicon sequencing-direct (GRAS-Di^®^) libraries of each specimen were prepared according to the protocol of Hosoya et al. (2019). The final PCR products were pooled, purified using the MiniElute PCR Purification Kit (Qiagen, Hilden, Germany), and applied for pair-end (2 × 76 bp) sequencing on the Illumina NextSeq 500. Library preparation and sequencing were done by Bioengineering Lab. Co., Ltd., under a license agreement, as GRAS-Di^®^ is patented by the Toyota Motor Corporation (Aichi, Japan) (patent ID P2018-42548A). The sequencing generated ~160 M (12.17 Gb) raw reads. These were trimmed in TrimGalore v. 0.6.4 (Krueger, 2019) to remove residues of indexes, nucleotides with quality ≤ Q20, and reads ≤ 20 bp after trimming. In total, 64.81 M reads in pairs (675,154 reads per sample on average) with a total length of 9.67 Gb (100.72 Mb per sample on average) were retained after trimming (Supplementary Table 2). Trimmed reads of each sample were mapped onto reference assembly using Bowtie2 v. 2.4 (Langmead and Salzberg, 2012) allowing no mismatches in the read seed (-N 0). The overall mapping rate was 92.2% with 74.9% of reads mapped only once (Supplementary Table 2). Sorted BAM files were created using samtools v. 1.10 (Danecek et al., 2021). Variants were called in bcftools v. 1.10.2 (Danecek et al., 2021) by *mpileup* and *call* commands using multiallelic-caller and variants-only flags (-mv). Filtering of SNPs was performed in vcftools v. 0.1.16 (Danecek et al., 2011) removing indels (--remove-indels), SNPs with quality ≤ 30 (--minQ 30), SNPs not in HWE (--hwe 0.05), and genotypes with depth ≤ 10 (--minDP 10). Minor allele frequency was set to 0.05 (--maf 0.05), retaining 5,853 SNPs of 368,265 raw variants. Genotypes were phased, and both female and male linkage maps were constructed in TMap v. 1.1 (Cartwright et al., 2007). All segregating markers that showed polymorphism in at least one parent were used. The ratio of marker segregation was calculated by chi-squared test. Markers showing significantly distorted segregation (*p*-value < 0.001) were excluded from the map construction. A minimum logarithm of odds (LOD) threshold of 5.0 was selected to assign markers to 24 linkage groups. Recombination rates were calculated by the multipoint-likelihood maximization, and map distances were converted by Kosambi mapping function. A total of 852 of 1,332 polymorphic loci were assigned to 24 linkage groups covering a total length of 1,554.7 cM of the *E. affinis* genome (Supplementary File 1).

To obtain haploid linkage evidence, interspecific hybrids of *E. affinis* and *T. orientalis* were produced. Briefly, eggs of a single *E. affinis* female were mixed with cryopreserved sperm of *T. orientalis* provided by the Aquaculture Research Institute, Kindai University, Japan. Seawater was immediately added to activate gametes and induce fertilization. After 1–2 h, eggs with proceeding cleavage were selected and transferred to the hatching tank at 24°C. After 36–40 h, 202 individuals that hatched or died after reaching somite formation were separately transferred to a 1.5-ml tube and stored in 100% ethanol. DNA was extracted from parents and F1 hybrids using a NucleoSpin Tissue XS kit (Macherey-Nagel, Düren, Germany). A sequencing library was prepared from 135 specimens, which provided sufficient DNA for use of the Nextera DNA Library Preparation Kit and Nextera Index Kit (Illumina) following the manufacturer's protocols. The library was sequenced on two lines of Illumina HiSeq X sequencing system. The sequencing resulted in 2,274 M sequencing reads with a total of 342 Gb. Reads were mapped onto a reference obtained by combining *E. affinis* scaffold-level assembly and *T. orientalis* genome (Suda et al., 2019) using BWA mem v. 0.7.15 (Li and Durbin, 2009). Mapped reads were sorted in samtools v. 1.10 (Danecek et al., 2021), and variants were called in bcftools v. 1.10.2 (Danecek et al., 2021) by *mpileup* and *call* commands. Linkage evidence of scaffolds was obtained through linkage analysis of hybrids in SELDLA v. 2.0.9 (Yoshitake et al., 2018) using 13,403,357 SNPs specific to *E. affinis*.

Female and male linkage maps and scaffold linkage evidence were transformed to BED files and merged. Pseudochromosomes were then reconstructed using ALLMAPS v. 1.1.7 from the JCVI utility libraries v. 0.7.5 (Tang et al., 2015) with inter-scaffold gaps set to a fixed size of 100 Ns. The package was used to merge bad files and to anchor, order, and orient genomic scaffolds using default parameters. Overall, 387 scaffolds with total length of 685.79 Mb (90.7% of scaffold-level assembly) were anchored onto 24 pseudochromosomes leaving 10,213 scaffolds of total length 72.42 Mb unplaced. Only two unplaced scaffolds had length > 1 Mb (Supplementary Figure 1). The final assembly contained 10,237 scaffolds of 758.24 Mb (19.96 Mb gaps), N50 = 29.18 Mb, and longest scaffold = 35.73 Mb (Table 1).

**Table 1:** Descriptive statistics of kawakawa *Euthynnus affinis* genome assembly, transcriptome assembly, repetitive DNA annotation, gene prediction, and functional annotation with completeness assessment results.

**Genome assembly**	
Number of sequences	10,237
Total length (bp)	758,243,246
N50 (bp)	29,176,746
Max scaffold length (bp)	35,734,308
GC content (%)	39.78
Gaps (bp)	19,960,513
BUSCO (%)	C = 97.1; S = 96.1; D = 1.0; F = 1.0; M = 1.9
**Transcriptome assembly**	
Number of sequences	49,510
Total length (bp)	94,582,375
N50 (bp)	3,622
Max contig length (bp)	56,879
BUSCO (%)	C = 91.7; S = 90.3; D = 1.4; F = 1.3; M = 7.0
**Repeat annotation**	
SINEs (bp)	1,061,850 (0.14%)
LINEs (bp)	22,627,927 (2.98%)
LTR elements (bp)	6,260,411 (0.83%)
DNA transposons (bp)	44,033,659 (5.81%)
Small RNA (bp)	570,727 (0.08%)
Satellites (bp)	101,725 (0.01%)
Simple repeats (bp)	19,817,678 (2.61%)
Low complexity (bp)	3,166,036 (0.42%)
Unclassified (bp)	94,139,074 (12.42%)
Total (bp)	194,013,215 (25.59%)
**Gene annotation**	
Number of predicted genes	23,059
Mean length (bp)	
Gene	10,511
Exon	244
Intron	939
CDS	1,592
Mean exon per gene	10
% of genome covered by genes	32.0
% of genome covered by CDS	4.8
BUSCO	C = 89.1; S = 87.8; D = 1.3; F = 3.4; M = 7.5
Functionally annotated total	21,313 (92.4%)
Swissport	19,750 (85.6%)
trEMBL	21,310 (92.4%)
NCBI NR	21,077 (91.4%)
InterPro	17,718 (76.8%)

*BUSCO = benchmarking universal single copy orthologs; C = complete; S = complete and single copy; D = complete and duplicated; F = fragmented; M = missing; LINEs = long interspersed nuclear elements; LTR = long terminal repeat; SINEs = short interspersed nuclear elements*.

### mRNA Sequencing and Transcriptome Assembly

Total RNA was extracted from eight tissues (brain, liver, kidney, ovary, testis, spleen, gill, muscle, and intestine) using TRIzol (*Invitrogen*, Carlsbad, CA, USA) and the NucleoSpin^®^ RNA Plus extraction kit (Macherey-Nagel, Düren, Germany) following the manufacturer's protocols for each tissue. RNA extracts were quantified using a NanoPhotometer N50 (Implen, München, Germany) and subsequently combined in equimolar quantities into a single pool for sequencing. RNA sequencing library was prepared by MGIEasy RNA Directional Library Prep Set (MGI Tech Co Ltd.). Pair-end (2 × 150 bp) sequencing was performed on a DNBSEQ-G400 sequencer (MGI Tech Co Ltd.). All procedures were conducted according to the manufacturer's protocols. The sequencing generated ~682 M (102.53 Gb) raw reads. Quality metrics for sequencing reads were initially examined in FastQC v. 0.11.9. (Andrews, 2020). Rare, possibly erroneous, k-mers were removed in Rcorrector v. 1.0.4 (Song and Florea, 2015) with default parameters, and adapters and low-quality bases were trimmed in TrimGalore v. 0.6.4 (Krueger, 2019) with parameters --length 36 -q 5 --stringency 3 -e 0.1 retaining ~614 M (92.18 Gb) pair-end reads. The FastQC results revealed deviation from normal distribution of GC content, and a high number of overrepresented sequences, possibly due to incomplete polyA capture during library preparation. Thus, trimmed reads were mapped against ribosomal RNA (rRNA) sequence database SILVA release 128 (Quast et al., 2012) using Bowtie2 v. 2.4 (Langmead and Salzberg, 2012) with parameters --nofw --quiet -D 20 -R 3 -N 0 -L 20 -i S,1,0.50 to remove rRNA contaminants. The 359.6 M (53.9 Gb) reads that did not map (--un-conc-gz) to the SILVA database were processed for *de novo* assembly in Trinity v. 2.11.0 (Grabherr et al., 2011) with default k-mer size 25 and --SS_lib_type RF --min_contig_length 300 flags. Initial transcriptome assembly resulted in 271,656 contigs of 242.43 Mb, N50 = 1.20 Kb. These were transferred to super transcripts (Davidson et al., 2017) by Trinity's v. 2.11.0 Trinity_gene_splice_modeler.py, and further redundancy was reduced by Bellerophon pipeline v. 1.0 (Kerkvliet et al., 2019), removing minimally expressed (transcripts per million cut off = 1) and highly identical (95%) contigs (CDHIT-EST -c 0.95). Final transcriptome assembly consisted of 49,510 contigs of 94.58 Mb, N50 = 3.62 Kb, with the longest contig = 56.88 Kb (Table 1).

### Repeat and Gene Annotation

**Figure 1:**
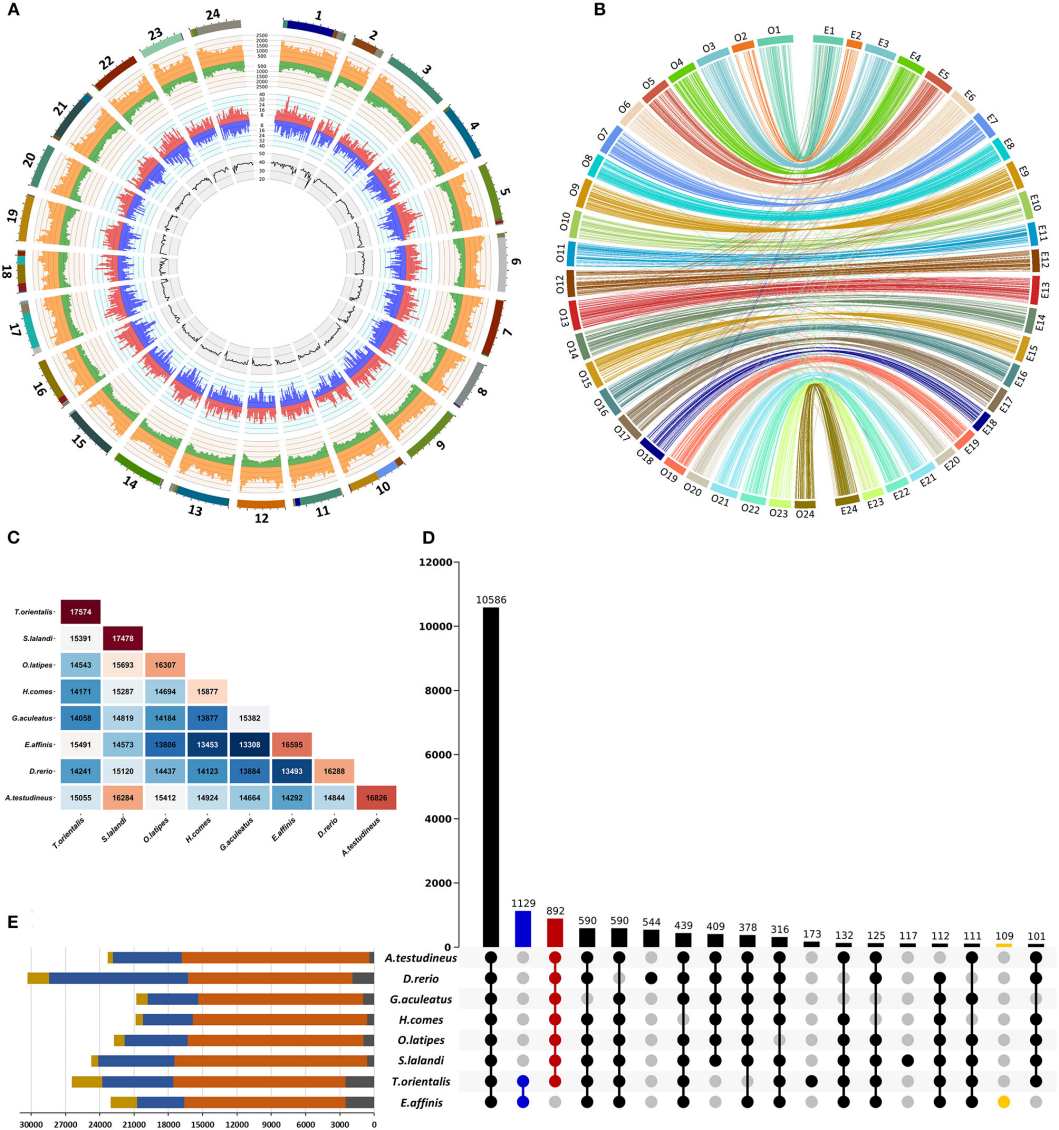
Characterization of the draft genome assembly of kawakawa *Euthynnus affinis*. **(A)** Summary of the genome annotation of kawakawa *Euthynnus affinis*. The tracks from inside to outside: GC content (%), negative-strand gene abundance (blue), positive-strand gene abundance (red), negative strand repetitive DNA abundance (green), positive-strand repetitive DNA abundance (orange), 24 pseudochromosomes (colors within each pseudochromosome denote different scaffolds). Window size = 1 Mb. **(B)** Chromosome level synteny between kawakawa *Euthynnus affinis* (right) and Japanese rice fish *Oryzias latipes* (left) based on 2,989 single-copy orthologs. **(C)** Number of orthogroups shared between each species pair of eight fish. **(D)** UpSet plot of intersections between orthogroups in different species. The bars show the number of common orthogroups for a given species or a group of species (dots connected by lines below the *x*-axis). In total, 10,586 orthogroups are common to all species, while 1,129 orthogroups are unique to *E. affinis* and *T. orientalis* (blue). *Euthynnus affinis–*specific orthogroups (109) are shown in yellow. The species lacks 892 orthogroups found in all other species (red). Intersections with fewer than 100 orthogroups are not shown. **(E)** Orthogroup size and proportion of genes assigned to orthogroups per species. Yellow = number of genes; blue = number of genes in orthogroups; orange = number of orthogroups containing a given species; gray = number of unassigned genes.

A *de novo* repeat library was generated using RepeatModeler v. 2.0.1 (Flynn et al., 2020) and MITE Tracker v. 1.0.0 (Crescente et al., 2018) with default parameters. The genome was then screened for repeats and low complexity regions by RepeatMasker v. 4.1.1 (Smit et al., 2015) in two runs using (i) *de novo*-generated repeat library and (ii) a Dfam database of interspersed repeats, release 3.3 (Storer et al., 2021). Results of the runs were analyzed together to generate final non-redundant repeat annotation. Repetitive regions accounted for 25.59% (194.01 Mb) of genome assembly (Figure 1A; Table 1). These included 12.42% unclassified repeats, 3.95% retrotransposons, 5.81% DNA transposons, 0.08% small RNAs, 0.01% satellites, 2.61% simple repeats, and 0.42% low complexity regions (Supplementary File 2).

Gene models were predicted in MAKER v. 3.01.03 (Holt and Yandell, 2011) in three successive runs. Prior to the first run, complex repeats were retrieved from the repeat annotation file and submitted to MAKER as pre-identified repeat elements (rm_gff) while still enabling the software to identify and soft mask simple repeats internally (Card, 2017). In this approach, complex repeats are hard masked so that they do not confound the ability to identify coding genes, while simple repeats remain available for inclusion in gene annotations, as many protein-coding genes contain runs of low-complexity sequence (Toll-Riera et al., 2011). During the first run, the *E. affinis* transcripts were aligned to the genome by BLASTN (Camacho et al., 2009) and protein sequences of *Danio rerio, Gasterosteus aculeatus, Hippocampus comes, Oreochromis niloticus, Oryzias latipes, Seriola dumerili, Sparus aurata*, and *Takifugu rubripes* from the Ensembl database v. 103 (Yates et al., 2019) along with *Thunnus orientalis* (Yasuike et al., 2016) by BLASTX (Camacho et al., 2009). Subsequently, BLAST hits were polished by Exonerate v. 2.4.7. (Slater and Birney, 2005) est2genome and protein2genome. All filtering statistics for BLAST and Exonerate were as the default by MAKER. The second and third runs of MAKER utilized gene models from the first, followed by the second, runs to train *ab initio* gene prediction tools SNAP v. 2013-02-16 (Korf, 2004) and Augustus v. 3.3.3. (Stanke and Waack, 2003). This bootstrap process allows to iteratively improve the performance of *ab initio* gene predictors as they require existing gene models on which to base prediction parameters. SNAP was retrained using gene models with an annotation edit distance (Holt and Yandell, 2011) (AED) ≤ 0.25 and amino acid length of ≥ 50. BUSCO v. 5.1.2 (Simao et al., 2015) with --long argument and actinopterygii_obd10 lineage dataset was used to retrain Augustus using genomic regions of RNA annotations from the previous run including an additional 1,000 bp on each side as input file. Both SNAP and Augustus were run with default parameters specified in MAKER. Only gene models with AED <0.5 were retained in the final annotation set.

For functional annotation of predicted genes, predicted protein sequences were mapped against UniProtKB/Swiss-Prot (The UniProt Consortium, 2021) and NCBI non-redundant (O'Leary et al., 2016) protein databases using BLASTP (Camacho et al., 2009) with an e-value threshold of 1e−6. Additionally, protein motifs, domains, and signatures were annotated using Interproscan v. 5.48 (Jones et al., 2014), and Gene Ontology (GO) terms were obtained from the corresponding InterPro entry. Kyoto Encyclopedia of Genes and Genomes Orthologs (KOs) were assigned to predicted proteins using KofamKOALA (Aramaki et al., 2019) with an e-value threshold of 1e−3.

In total, 23,059 putative genes spanning 32.0% of the genome were predicted (Figure 1A; Table 1). We found 21,313 (92.4%) predicted genes to match at least one of the databases (Table 1), and at least one GO and/or KO term was retrieved for 11,429 and 14,796 predicted genes, respectively.

## Data Validation

To validate the structural accuracy of the genome assembly and transcriptome assembly, Illumina and DNBSEQ pair-end sequencing reads were mapped back to the draft genome and transcriptome, respectively, using Bowtie2 v. 2.4 (Langmead and Salzberg, 2012) with default parameters. A total of 98.59% of the Illumina reads mapped to the genome with 97.4% of bases being covered >5 times. Mapping rate of clean DNBSEQ reads mapped to the transcriptome was 91.14% with 93.77% of bases being covered >5 times.

Completeness of both assemblies was accessed by two approaches. First, a total of 227 (97.42%) and 230 (98.71%) core vertebrate genes (CVGs) from the complete set of 233 CVGs (Hara et al., 2015) were identified in the genome and the transcriptome, respectively, by gVolante (Nishimura et al., 2017). Then, BUSCO v. 5.1.2 (Simao et al., 2015) was used to assess the presence of 3,640 actinopterygian single-copy orthologs (actinopterygii_odb10 lineage dataset) in both assemblies. In the genome, 3,534 (97.1%) single-copy orthologs were identified, of which 3,498 (96.1%) were complete and single copy. In the transcriptome, 3,338 (91.7%) single-copy orthologs were identified, of which 3,288 (90.3%) were complete and single copy (Table 1).

To verify the accuracy of the scaffold arrangement in 24 pseudochromosomes, the genomic locations of single-copy orthologs in *E. affinis* and *O. latipes* were compared and visualized in shinyCircos v. 1.0 (Yu et al., 2018). Only unduplicated orthologs co-identified in both species by BUSCO v. 5.1.2 (Simao et al., 2015) were used for comparison.

In summary, 2,561 of 2,989 single-copy orthologs were localized on the same chromosomes in both species (Figure 1B), revealing the high consistency of their genomes. Based on this result, the first 24 scaffolds of *E. affinis* genome assembly were numbered in concordance with *O. latipes* chromosomes.

The predicted gene set was assessed for completeness by the method used for genome and transcriptome assembly. The predicted gene set contained 215 (92.3%) of 233 CVGs (Hara et al., 2015) and 3,245 (89.1%) of 3,640 actinopterygian single-copy orthologs (Table 1). The predicted genes were clustered with those of seven fish species *A. testudineus, D. rerio, G. aculeatus, H. comes, O. latipes, S. lalandi*, and *T. orientalis* in OrthoFinder2 v. 2.3.8 (Emms and Kelly, 2015) to identify orthologous groups, i.e., a set of genes descended from a single gene in the last common ancestor (Emms and Kelly, 2015). The results were visualized in TBtools (Chen et al., 2020) and ggplot2 (Wickham, 2016).

In total, 181,780 of 192,254 genes from eight species were clustered into 21,003 orthogroups with 10,586 orthogroups being shared by all species (Figure 1D). Of 23,059 predicted genes of *E. affinis*, 20,749 were assigned to 16,595 orthogroups (Figures 1C,E) with 109 (311 genes) being unique to *E. affinis*. Species of the Thunnini tribe, *E. affinis* and *T. orientalis*, possessed 1,129 unique orthogroups (Figure 1D). In contrast, 892 orthogroups not detected in *E. affinis* were identified in all other species. This suggests that, although the genes belonging to these orthogroups were not captured during gene annotation, they are likely present in the *E. affinis* genome.

The present dataset was confirmed to be (i) accurate; (ii) sufficiently complete by current standards; (iii) consistent with genomic recourse of other closely related species; and (iv) reliably reusable for cooperative applications within the Thunnini tribe as well as within the entire Pelagiaria clade.

## Phylogenetic Analyses

Single-copy orthologs were identified in the *de novo* assembled *E. affinis* genome and across genomes of *Danio rerio* (GCF_000002035.6) and 15 representative species of the Percomorpha clade: *Anabas testudineus* (GCF_900324465.2), *Brotula barbata* (GCA_900303265.1), *G. aculeatus* (GCA_000180675.1), *Macroramphosus scolopax* (GCA_901007825.1), *O. niloticus* (GCF_001858045.2), *O. latipes* (GCA_002234675.1), *Paralichthys olivaceus* (GCA_001904815.2), *Periophthalmus magnuspinnatus* (GCF_009829125.1), *S. lalandi* (GCA_003054885.1), *S. aurata* (GCF_900880675.1), *Syngnathus acus* (GCF_901709675.1), *Thalassophryne amazonica* (GCA_902500255.1), *T. albacares* (GCA_900302625.1), *T. orientalis* (GCA_009176245.1), and *T. thynnus* (GCA_003231725.1) by BUSCO v. 3.0.2 (Simao et al., 2015). A total of 1,178 complete unduplicated BUSCO genes identified across genomes of all the above species were separately aligned in MAFFT v. 7.475 (Katoh and Standley, 2013) using the BLOSUM62 matrix of substitutions (--bl 62). Each alignment was trimmed in trimAl v. 1.4.1 (Capella-Gutiérrez et al., 2009) to remove sites of unclear homology using the heuristic method *automated1*. The resulting alignments were concatenated by catsequences v. 1.3. (Creevey, 2021) (Supplementary File 3), and the species tree was inferred in IQ-TREE v. 2.0.3 (Nguyen et al., 2015) letting ModelFinder (Kalyaanamoorthy et al., 2017) select the optimal substitution model for each partition prior to running the tree interface with 1,000 ultrafast bootstrap replicates (Hoang et al., 2018) and 1,000 replicates for the Shimodaira-Hasegawa-like approximate likelihood ratio test (SH-aLRT) (Guindon et al., 2010). The divergence time was estimated with MCMCTree in the package PAML 4.9j (Yang, 1997) using parameters with independent clock rates. Calibration divergence times of *A. testudineus* from *S. lalandi* (91–102 million years ago [Mya]), *T. orientalis* from *O. latipes* (106–144 Mya), and *D. rerio* from Percomorpha (206–252 Mya) obtained from TimeTree database (Kumar et al., 2017) were used as time scales to estimate the divergence time of *E. affinis* from other percomorph species. The final tree was drawn in FigTree v. 1.4.4 (Rambaut, 2018).

Observed phylogenetic relationships were consistent with recent studies of phylogeny of the Percomorpha clade (Sanciangco et al., 2016; Friedman et al., 2019). The divergence time of *E. affinis* from a common tuna ancestor was inferred to be ~46.9 Mya (Supplementary File 4; Supplementary Figure 2). This is more than twice the age estimated in the TimeTree database (Kumar et al., 2017) but in agreement with the most recent study by Friedman et al. (2019).

## Data Availability Statement

The datasets presented in this study can be found in online repositories. The names of the repository/repositories and accession number(s) can be found below: https://www.ddbj.nig.ac.jp/, BPLY01000001 - BPLY01010237, ICRR01000001 - ICRR01049510, DRA012118, DRA012140, DRA012141, https://www.ncbi.nlm.nih.gov/, , https://figshare.com/, doi: 10.6084/m9.figshare.14937774.

## Ethics Statement

The animal study was reviewed and approved by Animal Care Committee of Ehime University.

## Author Contributions

MH and ES: conceptualization, writing—review and editing. MH, ES, TS, and KY: data curation. MH, ES, KY, and DS: formal analysis, investigation. MT, SA, and TM: funding acquisition. RG, MT, TM: project administration. TS, RG, TM, SA, and TI: resources. MH, ES, and KY: validation. MH and TS: visualization. MH and KY: writing original draft. MH: performed the majority of data analysis. DS, KY, TI, and SA contributed solely to tasks related to the production of interspecific hybrids and their subsequent analyses. All authors read and approved the final version of the manuscript.

## Funding

The study was financially supported by the Ministry of Education, Culture, Sports, Science and Technology of Japan (MEXT) under the Regional Innovation and Ecosystem Formation Program (FY2017-2021); Bio-oriented Technology Research Advancement, Naro (BRAIN) under the special scheme project on regional developing strategy (16818524); and the National University Corporation Ehime University Research Fellow Employment Support Expenses (2021) under the special scheme project on regional developing strategy (ZK39b).

## Conflict of Interest

The authors declare that the research was conducted in the absence of any commercial or financial relationships that could be construed as a potential conflict of interest.

## Publisher's Note

All claims expressed in this article are solely those of the authors and do not necessarily represent those of their affiliated organizations, or those of the publisher, the editors and the reviewers. Any product that may be evaluated in this article, or claim that may be made by its manufacturer, is not guaranteed or endorsed by the publisher.

## Supplementary Material

Supplementary Figure 1 Length distribution of kawakawa *Euthynnus affinis* genome assembly.

Supplementary Figure 2 Time-calibrated phylogeny of representative species of the nine major percomorph clades with zebrafish *Danio rerio* as outgroup.

Supplementary File 1 Genetic linkage information of each marker with its location in both scaffold level assembly and pseudochromosome level assembly.

Supplementary File 2 Major categories and quantities of repeat elements in *E. affinis* genome assembly.

Supplementary File 3 Nucleotide alignment of 1,179 single copy orthologs of *Euthynnus affinis, Danio rerio* and 15 representative species of Percomorpha clade.

Supplementary File 4 Phylogenetic tree and partition information giving position of each gene in the alignment and substitution model for each gene.

Supplementary Table 1 Results of genome characteristics estimate for three k-mers (19, 21, and 23).

Supplementary Table 2 Detailed data for all specimens used for genotyping by sequencing and linkage mapping. These include phenotype data, the number of sequencing reads and length of the data before and after trimming, and results of mapping of trimmed reads of each specimen to *E. affinis* genome assembly.
