# Supplementary figures and images for: Chromosome-Scale Genome Assembly and Transcriptome Assembly of Kawakawa Euthynnus affinis; A Tuna-Like Species
Source: Front Genet. 2021 Sep 20;12:739781. doi: 10.3389/fgene.2021.739781 (PMC8489456; doi:10.3389/fgene.2021.739781)

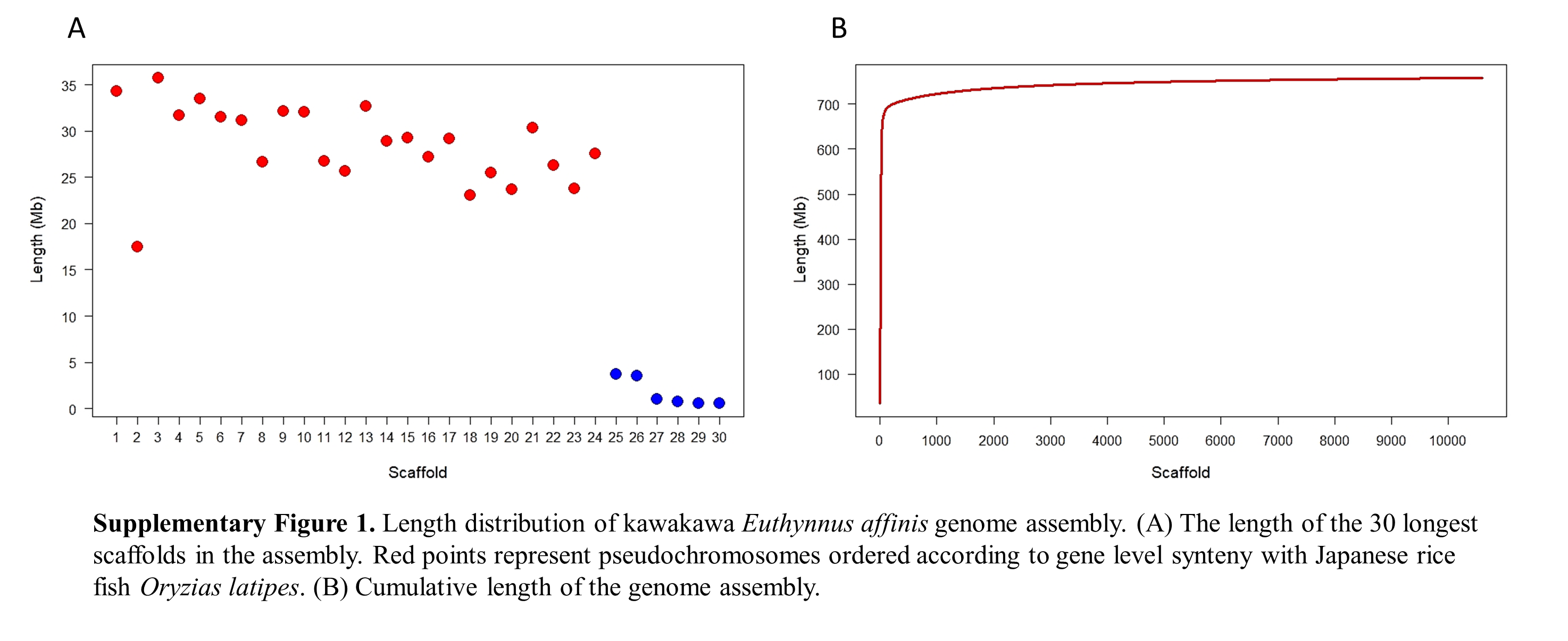

Supplement: Supplementary Figure 1 — Length distribution of kawakawa Euthynnus affinis genome assembly. [file Image_1.TIF]

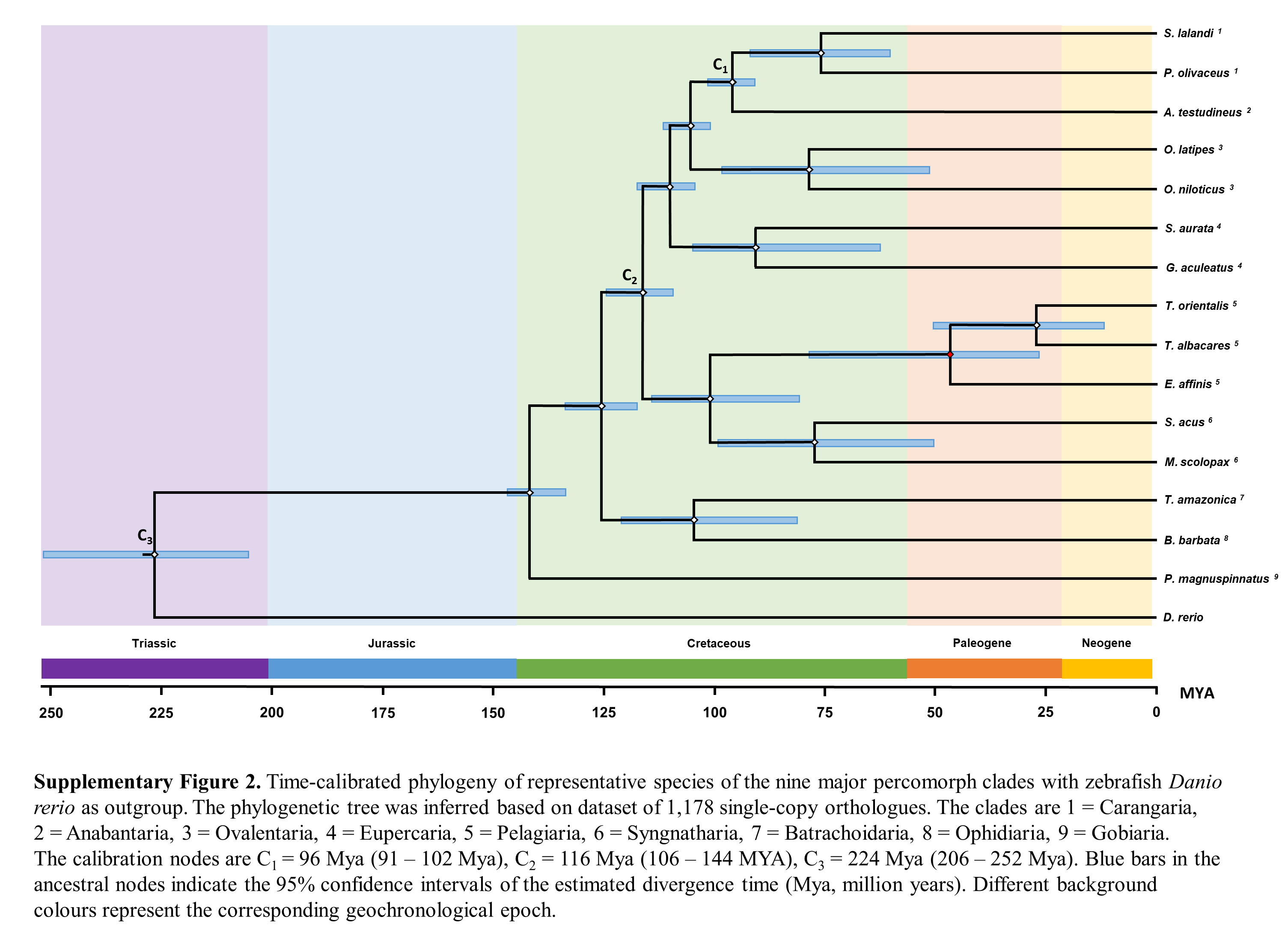

Supplement: Supplementary Figure 2 — Time-calibrated phylogeny of representative species of the nine major percomorph clades with zebrafish Danio rerio as outgroup. [file Image_2.TIF]
