# Supplementary material for: Chromosome-Scale Genome Assembly and Transcriptome Assembly of Kawakawa Euthynnus affinis; A Tuna-Like Species
Source: Front Genet. 2021 Sep 20;12:739781. doi: 10.3389/fgene.2021.739781 (PMC8489456; doi:10.3389/fgene.2021.739781)
Supplement: Supplementary File 3 — Nucleotide alignment of 1,179 single copy orthologs of Euthynnus affinis, Danio rerio and 15 representative species of Percomorpha clade. [file Data_Sheet_3.PDF]

Supplementary File 2. - Major categories and quantities of repeat elements in E. affinis genome assembly.

==

file name: Eaf.full_mask
sequences: 10237
total length: 758243246 bp (738282733 bp excl N/X-runs)
GC level: 39.76 %
bases masked: 194013215 bp (25.59 %)

==

	number of elements*	length occupied	percentage of sequence
Retroelements	128357	29950188 bp	3.95 %
SINEs:	8506	1061850 bp	0.14 %
Penelope	3435	644166 bp	0.08 %
LINEs:	93948	22627927 bp	2.98 %
CRE/SLACS	0	0 bp	0.00 %
L2/CR1/Rex	68347	15855050 bp	2.09 %
R1/LOA/Jockey	5060	1168148 bp	0.15 %
R2/R4/NeSL	1871	696795 bp	0.09 %
RTE/Bov-B	6852	1808759 bp	0.24 %
L1/CIN4	5108	1565859 bp	0.21 %
LTR elements:	25903	6260411 bp	0.83 %
BEL/Pao	1602	817959 bp	0.11 %
Ty1/Copia	35	50700 bp	0.01 %
Gypsy/DIRS1	13173	3129869 bp	0.41 %
Retroviral	3556	977627 bp	0.13 %
DNA transposons	280294	4403365 9bp	5.81 %
hobo-Activator	118559	18740535 bp	2.47 %
Tc1-IS630-Pogo	28140	5376673 bp	0.71 %
En-Spm	0	0 bp	0.00 %
MuDR-IS905	0	0 bp	0.00 %
PiggyBac	423	86039 bp	0.01 %
Tourist/Harbinger	17724	3694363 bp	0.49 %
MITEs	32034	4834941 bp	0.64 %
Other (Mirage, P-element, Transib)	8418	1625151 bp	0.21 %
Rolling-circles	9877	2659008 bp	0.35 %
Unclassified:	562619	94139074 bp	12.42 %
Total interspersed repeats:		168122921 bp	22.17 %
Small RNA:	5334	570727 bp	0.08 %
Satellites:	1070	101725 bp	0.01 %
Simple repeats:	508276	19817678 bp	2.61 %
Low complexity:	60657	3166036 bp	0.42 %

==

* most repeats fragmented by insertions or deletions have been counted as one element

The query species was assumed to be actinopterygii
RepeatMasker version 4.1.1 , default mode

run with rmblastn version 2.10.0+
