# Supplementary material for: Chromosome-Scale Genome Assembly and Transcriptome Assembly of Kawakawa Euthynnus affinis; A Tuna-Like Species
Source: Front Genet. 2021 Sep 20;12:739781. doi: 10.3389/fgene.2021.739781 (PMC8489456; doi:10.3389/fgene.2021.739781)
Supplement: Supplementary Table 1 — Results of genome characteristics estimate for three k-mers (19, 21, and 23). [file Table_1.DOCX]

| **k = 19** |  |  |
| --- | --- | --- |
| **property** | **min** | **max** |
| **Heterozygosity** | 0.67% | 0.67% |
| **Haploid Length** | 745.82 Mb | 746.30 Mb |
| **Repeat Length** | 170.24 Mb | 170.35 Mb |
| **Unique Length** | 575.58 Mb | 575.96 Mb |
| **Model Fit** | 92.71% | 94.38% |
| **Read Error Rate** | 0.71% | 0.71% |
| **Link** | <http://genomescope.org/analysis.php?code=XMMq5dqtpjkusw8bMQTP> | |
|  |  |  |
|  |  |  |
| **k = 21** |  |  |
| **property** | **min** | **max** |
| **Heterozygosity** | 0.68% | 0.68% |
| **Haploid Length** | 750.40 Mb | 751.88 Mb |
| **Repeat Length** | 144.06 Mb | 144.15 Mb |
| **Unique Length** | 607.34 Mb | 607.73 Mb |
| **Model Fit** | 93.25% | 94.55% |
| **Read Error Rate** | 0.70% | 0.70% |
| **Link** | <http://genomescope.org/analysis.php?code=jowYCBx3kHtNo1cKJcvp> | |
|  |  |  |
|  |  |  |
| **k = 23** |  |  |
| **property** | **min** | **max** |
| **Heterozygosity** | 0.67% | 0.68% |
| **Haploid Length** | 755.32 Mb | 755.80 Mb |
| **Repeat Length** | 136.92 Mb | 137.00 Mb |
| **Unique Length** | 618.38 Mb | 618.78 Mb |
| **Model Fit** | 93.49% | 94.65% |
| **Read Error Rate** | 0.67% | 0.67% |
| **Link** | <http://genomescope.org/analysis.php?code=GEAnwhVz6LG3v3Wp41kX> | |

**Supplementary Table 1.** Results of genome characteristics estimate for three k-mers (19, 21, and 23).
